# Resilience in Urban Pharmacies: A Mixed-Methods Model for Performance and Patient Satisfaction During Crises

**DOI:** 10.5812/ijpr-166647

**Published:** 2025-12-22

**Authors:** Javad Shamsaee, Farzad Peiravian, Samane Jahanabadi, Mohammad Peikanpour

**Affiliations:** 1 Department of Pharmacoeconomics and Pharma Management, School of Pharmacy, Shahid Beheshti University of Medical Sciences, Tehran, Iran; 2 Department of Pharmacology and Toxicology, Faculty of Pharmacy, Iran University of Medical Sciences, Tehran, Iran; 3 Department of Pharmacology and Toxicology, Faculty of Pharmacy, Shahid Sadoughi University of Medical Sciences, Yazd, Iran

**Keywords:** Resilience, Pharmacy Performance, Patient Satisfaction, Pharmacy

## Abstract

**Background:**

Urban pharmacies are essential for sustaining access to medicines and healthcare services during crises, particularly during the COVID-19 pandemic. The role of pharmacies and the services provided by pharmacists during crises have been relatively underexplored.

**Objectives:**

This study aims to propose a model for reforming pharmaceutical policy to ensure the continuity of pharmacist-led services during crises.

**Methods:**

In this cross-sectional study, a mixed-methods approach was employed. In the qualitative phase, semi-structured interviews were conducted with 15 experienced pharmacy experts, and thematic analysis was carried out. In the quantitative phase, structured questionnaires measuring resilience, performance, and satisfaction were distributed to 330 randomly selected pharmacies in Tehran, analyzed using SPSS version 25.

**Results:**

In the qualitative phase, the research identified three main themes, including resilience, performance, and patient satisfaction, along with nine subthemes. In the quantitative phase, significant positive correlations were found between pharmacy resilience and performance (R = 0.642), resilience and patient satisfaction (R = 0.496), and a moderate correlation between patient satisfaction and performance (R = 0.334). Partial correlation analysis confirmed the robustness of this model while accounting for potential confounders. Based on the results, a significant difference was observed between manager’s gender and pharmacy resilience, performance, and satisfaction of patient. Patient satisfaction was significantly higher in pharmacies managed by male directors compared to those managed by female directors (P < 0.05). A statistically significant association was found between drug availability and pharmacy performance (P < 0.05).

**Conclusions:**

Pharmacy resilience emerged as a crucial determinant of operational stability and patient-centered outcomes during health emergencies. The proposed model provides a practical framework for strengthening pharmacy readiness, response, recovery, and adaptive growth, with potential applicability to broaden health system resilience efforts. Policymakers and pharmacy managers are encouraged to integrate resilience-building strategies to ensure sustainable performance and patient satisfaction during future crises.

## 1. Background

Urban pharmacies are increasingly recognized as critical nodes in public health infrastructure, particularly during times of crisis, such as the COVID-19 pandemic. Pharmacies’ workloads have increased rapidly with the addition of responsibilities including delivering public health interventions, managing medicine supply, and implementing policy changes (1). Despite global efforts to return to normal conditions after the pandemic, there are concerns about subsequent waves of the pandemic. The COVID-19 pandemic has imparted critical lessons to society, underscoring the significance of resilience across various components of the essential goods supply chain, particularly in the pharmaceutical sector.

Resilience can be characterized as the capacity to recover from challenges and is recognized as a dynamic process that changes in accordance with both internal and external factors. Resilience within the pharmacy profession can be examined at both the individual level of pharmacists and the broader organizational level. It has been proposed that achieving a high level of professional resilience among individuals requires considerable support from the organizational level (2, 3). Today, literature on resilience has developed in the fields of macroeconomics, risk management, supply chain management, and disaster management. Resilience has found a special place in various management sectors such as health and the pharmaceutical supply chain (4). The need for pharmacists and pharmacy services is essential and tends to increase during times of crisis such as pandemics or widespread civil unrest (5). Recently, the concept of resilience has gained increasing attention in healthcare systems due to its central role in ensuring continuity of services under crisis conditions. Particularly within community pharmacy practice, resilience enhances the ability to adapt workflows, sustain medicine availability, and meet patient needs despite disruptions in supply chains or workforce shortages (6, 7). Resilient health systems demonstrated a greater capacity to control disease outbreaks, recover to baseline function faster, and be informed by lessons learnt during the crisis (8). This has led to a growing recognition that pharmacies, as both clinical and commercial entities, require tailored resilience frameworks that account for their unique operational, regulatory, and interpersonal dynamics (3, 5).

Moreover, resilience enables organizations to more effectively adjust their overall performance in response to unforeseen circumstances, allowing them to navigate a dynamic environment and evolving consumer behaviors (9). Prior research suggests that companies implementing a resilient approach possess the necessary tools to effectively manage disturbances, leading to enhanced performance outcomes (10, 11). A resilient organization should prioritize comprehending the entirety of the situation and commit to the ongoing enhancement of its products and services, overall performance, long-term sustainability, and customer satisfaction and loyalty (12). Consequently, it is imperative for organizations to strengthen their resilience strategies to boost their overall efficiency and increase customer loyalty, particularly after the COVID-19 pandemic, which negatively affected the majority of micro, small, and medium-sized enterprises (MSMEs) (13). In healthcare, patient satisfaction has emerged as a crucial indicator in evaluating the quality of pharmacy services, serving both as a key measure of service effectiveness and a vital driver of continuous improvement in healthcare delivery systems. Patient satisfaction, a complex and multidimensional concept, is becoming increasingly essential amid the evolving structures of professional healthcare systems (14, 15). Customer satisfaction is influenced by infrastructure, product availability, medication information, and trust in the pharmacy (16).

**Figure 1.**
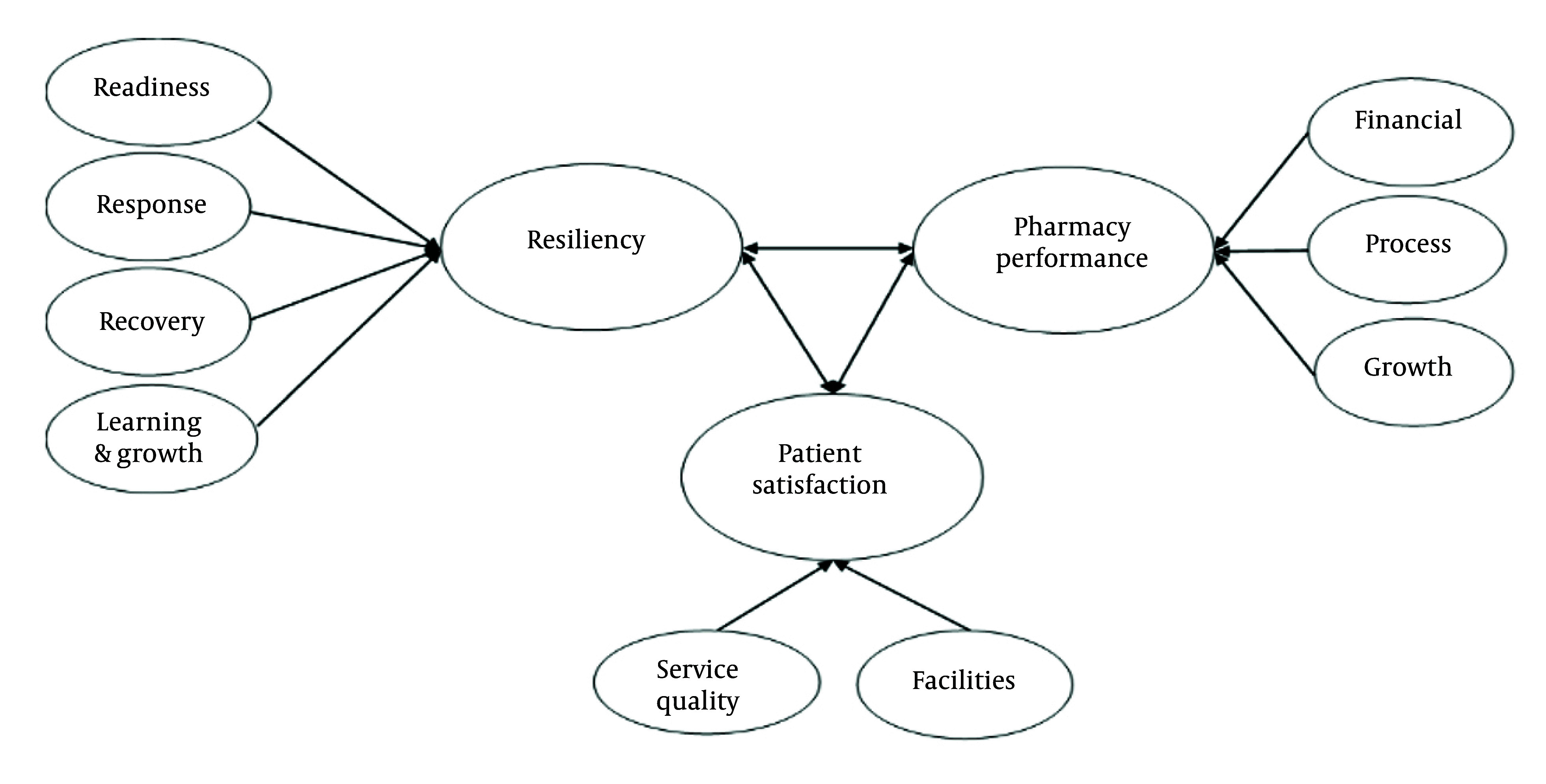
Conceptual framework and factors influencing research variables

Most of the previous literature has highlighted the considerable beneficial effect of resilience on organizational performance and survival, particularly in the wake of the COVID-19 pandemic (13), but its impact on pharmacy-specific performance indicators and patient satisfaction remains underexplored. Based on the conducted literature review, the conceptual framework of the study is summarized as follows (Figure 1). 

## 2. Objectives

Considering the special position of urban outpatient pharmacies in improving the resilience of the pharmaceutical system against unforeseen shocks, this study examines the relationship between various dimensions of resilience and the performance of urban outpatient pharmacies. The goal of this research is to provide solutions to improve service levels, increase patient satisfaction by making pharmacies more resilient in crisis situations, and develop a resilience model in urban outpatient pharmacies during the COVID-19 pandemic. Based on the foregoing, three hypotheses are proposed: H1. Resilience is correlated with performance of pharmacy. H2. Resilience is correlated with patient satisfaction in pharmacy. H3. Pharmacy performance is correlated with patient satisfaction in pharmacy.

## 3. Methods

This research, using a mixed-methods approach (qualitative-quantitative), examined the relationship between resilience, performance, and patient satisfaction of urban outpatient pharmacies in dealing with crises, particularly the COVID-19 pandemic. The qualitative phase aimed to identify the dimensions and indicators of resilience, performance, and patient satisfaction relevant to pharmacies and to gain a deeper understanding of existing challenges and solutions. Meanwhile, the quantitative phase focused on assessing the level of resilience, performance, and patient satisfaction of pharmacies, as well as statistically analyzing the relationship between these three variables.

### 3.1. Qualitative Phase

Based on an extensive literature review and semi-structured interviews with experts, the dimensions of pharmacy resilience, pharmacy performance, and patient satisfaction were identified and further developed. Semi-structured interviews were conducted with 15 experts who had at least five years of experience managing public and private pharmacies. Each interview lasted an average of 45 minutes. Qualitative thematic analysis was performed. To achieve this objective, the involvement of independent coders played a crucial role in capturing the themes derived from the data. In this study, coding was conducted by two members of the research team using Excel 2019. The insights provided were consolidated, incorporating feedback from supervisors. Primary codes, secondary codes, categories, and themes were then extracted. Ultimately, this led to the development of a research measurement tool in the form of questionnaires for pharmacy managers and patients. During the study, two questionnaires were developed. One was completed by pharmacy managers, encompassing dimensions of resilience and pharmacy performance. The second questionnaire, focusing on patient satisfaction, was completed by two patients visiting each pharmacy.

### 3.2. Quantitative Phase

A cross-sectional survey was carried out in pharmacies during this stage through the collection of data through a self-administered questionnaire from February 2024 to July 2024 in Tehran. The questionnaires were distributed to pharmacies in five regions (North, South, East, West, and center).

The collected data were analyzed using descriptive indicators such as frequency, percentage, mean, standard deviation, and mean rank. Additionally, non-parametric statistical tests, including the Mann-Whitney U test and the Wilcoxon W test, were employed for statistical analysis. The data were analyzed using SPSS software (version 25). The relationships between various dimensions were also examined using Spearman correlation coefficient tests, and ultimately, the proposed model was tested.

## 4. Results

### 4.1. Qualitative Phase

The interviewees primarily consisted of experts with at least five years of experience managing both public and private pharmacies. The range of pharmacy management experience among interviewees was between 5 and 45 years. The interviewees comprised both male and female participants, possessing either a pharmacy degree or a PhD in pharmacy. The findings identified resilience, pharmacy performance, and patient satisfaction as the main themes, which included four subthemes related to resilience, three subthemes related to pharmacy performance, and two subthemes related to patient satisfaction.

#### 4.1.1. Concept of Resilience in Pharmacy

Interview findings revealed that resilience in pharmacy practice is a multifaceted construct encompassing readiness, response, recovery, learning, and growth.

1. Readiness: System preparedness to withstand both anticipated and unanticipated events, as well as internal and external risks (17, 18).

2. Response: Rapid and effective response during disruptions to facilitate adaptation to the disturbance (17).

3. Recovery: Swift restoration to the previous normal state following the stabilization of conditions (18, 19).

4. Learning and growth: Achieving a superior position compared to pre-crisis conditions in relation to competitors (19).

#### 4.1.2. Concept of Performance in Pharmacy

Interview findings revealed that performance in pharmacy is a multifaceted construct encompassing financial performance, pharmacy process, and growth.

1. Financial performance: Improvement of financial indicators including sales, profit, and revenue, and employee satisfaction (20, 21).

2. Pharmacy process: Refining and redefining processes, flexibility, and promoting educational advancement (20).

3. Growth: Improvement of human and financial resources (20).

#### 4.1.3. Concept of Patient Satisfaction

Interview findings revealed that patient satisfaction is a multifaceted construct encompassing pharmacy facilities and service quality.

1. Pharmacy facilities: Include responsiveness, effective communication, trust, and reliability (22).

2. Service quality: Physical facilities and service upgrades (21).

### 4.2. Quantitative Phase

In this study, 450 community pharmacies in Tehran were randomly selected, and two questionnaires were distributed. The study received 330 responses from pharmacy managers and 660 from patients, resulting in a 73% response rate. The Likert scale included five points, where 1 represented the lowest value and 5 the highest value, for the respondents' answers.

The reliability of the questionnaire was acceptable with a Cronbach’s alpha of 0.7. Table 1 presents the result of the reliability evaluation for each aspect of the questionnaire. Upon completion of the qualitative phase and during the development of the measurement instrument, the face and content validity of the questionnaires were systematically evaluated. This process involved a panel of experts, including specialists in pharmaceutical economics and management, as well as experienced pharmacy managers. The evaluation employed the Content Validity Ratio (CVR) to assess each item across four dimensions: Necessity, clarity, simplicity, and relevance to the domain. Two items yielded CVR values below the established threshold, while all items demonstrated acceptable levels of Content Validity Index (CVI). Following the incorporation of expert feedback and recommended revisions, the finalized questionnaires, targeting pharmacy managers and patients, were prepared for implementation in the subsequent phase of the study.

**Table 1.** The Results of Reliability Assessment for Each Aspect in the Questionnaire

Aspect	Cronbach’s Alpha	n
**Readiness**	0.91	21
**Response**	0.83	27
**Recovery**	0.84	15
**Learning and growth**	0.78	57
**Financial**	0.88	15
**Process**	0.79	16
**Growth**	0.78	57
**Pharmacy facilities**	0.81	47
**Service quality**	0.90	716

The demographic data of the respondents indicated a normal distribution among the participants. The demographic characteristics of the studied participants, including gender, level of education, geographic location, job status, and purpose as well as history of pharmacy visits are summarized in Tables 2. and 3. 

**Table 2.** Summary of the Characteristics of the Respondents (Pharmacy Professionals)

Characteristics	Frequency (%)	P-Value ^a^		
		Resiliency	Patient Satisfaction	Pharmacy Performance
**Gender**				
Male	62.4	NS	< 0.05	NS
Female	37.6	NS	NS	NS
**Age**				
≤ 30 years	7.3	NS	NS	NS
31 - 40 years	32.3	NS	NS	NS
41 - 50 years	26.2	NS	NS	NS
51 - 60 years	11.3	NS	NS	NS
≥ 61 years	23.0	< 0.001	< 0.001	< 0.001
**Educational level**				
High school	0	NS	NS	NS
Diploma	20.1	NS	NS	NS
Bachelor	21.3	NS	NS	NS
Master	8.0	NS	NS	NS
Doctorates	50.6	NS	NS	NS
**Pharmacy responsibilities**				
Pharmacy founder	49.5	NS	NS	NS
Pharmacy manager	44.5	NS	NS	NS
Pharmacist	6.0	NS	NS	NS
**Pharmacy ownership**				
Yes	46.0	NS	NS	NS
No	54.0	NS	NS	< 0.05
**Location**				
North	17	< 0.001	< 0.001	< 0.05
South	13	NS	NS	NS
East	24	NS	NS	NS
West	21	< 0.001	< 0.001	< 0.05
Center	25	NS	NS	NS
**Pharmacy operating hours**				
Part-Time	4.0	NS	NS	NS
Daytime	74.5	NS	NS	NS
24 - hour pharmacy	21.5	NS	NS	NS

Abbreviation: Ns, not significant.

^a^ Only P < 0.05 is reported in this table.

**Table 3.** Summary of the Characteristics of the Respondents (Pharmacy Visitors)

Characteristics	Frequency (%)	P-Value ^a^		
		Resiliency	Patient Satisfaction	Pharmacy Performance
**Gender**				
Male	56.3	NS	NS	NS
Female	43.2	NS	NS	NS
**Age**				
≤ 30 years	24.6	NS	NS	NS
31 - 40 years	24.6	NS	NS	NS
41 - 50 years	14.5	NS	NS	NS
51 - 60 years	5.6	NS	NS	NS
≥ 61 years	30.6	NS	NS	NS
**Educational level**				
High school	7.7	NS	NS	NS
Diploma	49.2	NS	NS	NS
Bachelor	31.4	NS	NS	NS
Master	11.8	NS	NS	NS
Doctorates	0	NS	NS	NS
**Reason for selecting this pharmacy**				
Residential proximity	39.4	NS	NS	NS
Service satisfaction	20.6	NS	NS	NS
Medication availability	11.7	NS	NS	< 0.05
Residential proximity and service satisfaction	28.3	NS	NS	NS
**History of pharmacy visits**				
≤ 5 years	63	NS	NS	NS
6 - 10 years	28.8	NS	NS	NS
> 10 years	8.2	NS	< 0.05	NS

Abbreviation: Ns, not significant.

^a^ Only P < 0.05 is reported in this table.

Based on the results, a significant association was observed between the age of pharmacy managers and key outcome variables, including resilience, pharmacy performance, and patient satisfaction (P-value < 0.001; Table 2). As indicated in Table 2, the gender of the pharmacy manager had an impact on patient satisfaction outcomes, suggesting a statistically significant relationship between manager gender and patient satisfaction. Specifically, patient satisfaction was higher in pharmacies managed by male managers compared to those managed by female managers (P-value < 0.05). The analysis revealed statistically significant differences in resilience, pharmacy performance, and patient satisfaction based on geographic location, specifically in northern and western regions. Furthermore, pharmacy ownership status was positively associated with enhanced pharmacy performance. Pharmacies in which the manager did not hold ownership exhibited superior operational performance compared to those managed by owner-pharmacists (P-value < 0.05; Table 2). 

As shown in Table 3, a statistically significant association was found between the reason for visiting the pharmacy and its performance, with drug availability demonstrating a stronger effect than other factors such as service satisfaction or proximity to home (P-value < 0.05). Moreover, the history of pharmacy visits exceeding ten years was significantly associated with patient satisfaction (P-value < 0.05).

The relationship between resilience, pharmacy performance, and patient satisfaction was assessed using correlation analysis. Pearson’s correlation coefficient assumes that the data follow a normal distribution and that the relationship between variables is linear. This test determines both the direction and strength of the association between two variables. As shown in Table 4, the results indicate a significant positive correlation between resilience and patient satisfaction (R = 0.496; P-value < 0.001), and between resilience and pharmacy performance (R = 0.642; P-value < 0.001). Additionally, a moderate correlation was found between patient satisfaction and pharmacy performance (R = 0.334; P-value < 0.001).

**Table 4.** Pearson Correlation Coefficients and Significance Levels Among Study Variables

Spearman's rho	Resiliency	Patient Satisfaction	Pharmacy Performance
**Resiliency**			
Correlation coefficient	1.000	0.496 ^a^	0.642 ^a^
Sig. (2-tailed)	-	0.000	0.000
**Patient satisfaction**			
Correlation coefficient	0.496 ^a^	1.000	0.334 ^a^
Sig. (2-tailed)	0.000	-	0.000
**Pharmacy performance**			
Correlation coefficient	0.642 ^a^	0.334^a^	1.000
Sig. (2-tailed)	0.000	0.000	-

^a^ significance at the 0.01 level (2-tailed).

To assess the effect of a potential confounding variable, partial correlation analysis was employed. Partial correlation is a statistical technique used to measure the strength and direction of the relationship between two variables while controlling for the influence of one or more additional variables. By isolating the impact of confounders, this method enables the identification of the direct association between the target variables. As presented in Tables 4 and 5, a statistically significant positive and direct correlation was observed between resilience and patient satisfaction (R = 0.496), as well as between resilience and pharmacy performance (R = 0.542). Additionally, a modest correlation was found between patient satisfaction and pharmacy performance (R = 0.288).

**Table 5.** Pearson's Partial Correlation Coefficients (PCC) and Significance Levels Among Study Variables

Spearman's rho	Resiliency	Patient Satisfaction	Pharmacy Performance
**Resiliency**			
Correlation coefficient	1.000	0.496	0.542
Sig. (2-tailed)	-	0.000	0.000
**Patient satisfaction**			
Correlation coefficient	0.496	1.000	0.288
Sig. (2-tailed)	0.000	-	0.000
**Pharmacy performance**			
Correlation coefficient	0.542	0.288	1.000
Sig. (2-tailed)	0.000	0.000	-

Based on the study results, it was concluded that H1, H2, and H3 are accepted for the whole entire sample. The following Table 6 summarizes the testing of the research hypotheses.

**Table 6.** Research Hypotheses Testing

Hypothesis	Correlation	Partial Correlation	P-Value	Hypothesis Testing
Resilience → Pharmacy performance	0.642	0.542	0.000	accepted
Resilience → Patient satisfaction	0.496	0.496	0.000	accepted
Pharmacy performance → Patient satisfaction	0.334	0.288	0.000	accepted

**Figure 2.**
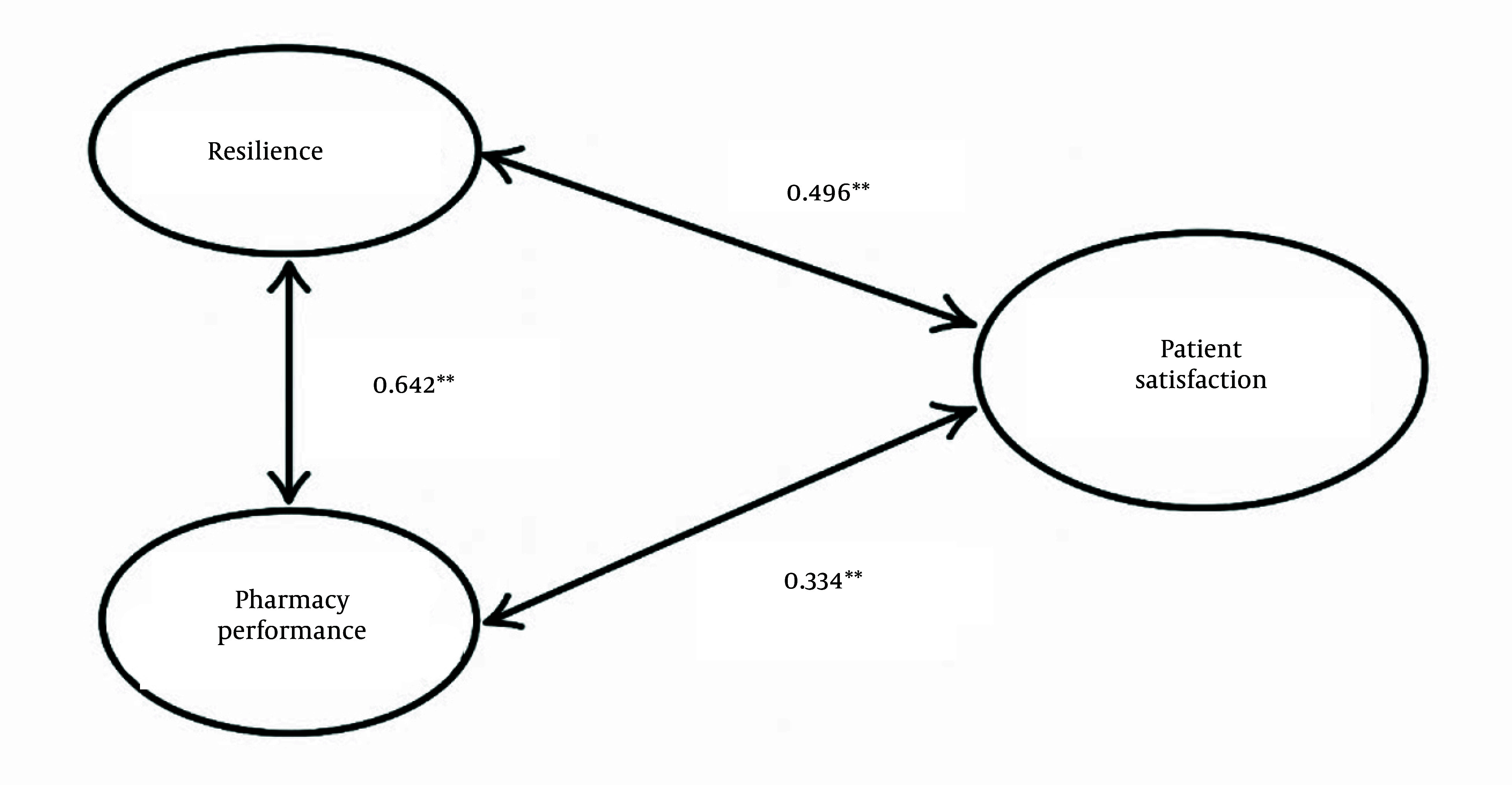
Research model results (** Correlation is significant at the 0.01 level (2-tailed)

A conceptual model was developed to evaluate the factors influencing the correlation between resilience and the performance of urban outpatient pharmacies during the COVID-19 pandemic (Figure 2). This model was derived from a literature review and expert consultations with senior pharmacy professionals. The extracted codes encompassed 94 items categorized under nine model-related indicators, addressing dimensions such as resilience, patient satisfaction, and pharmacy performance. Based on the complete questionnaires from pharmacy managers and clients, and the subsequent weighting of research components, a statistically significant and strong association was identified between resilience and pharmacy performance. Furthermore, meaningful correlations were also observed between patient satisfaction and both resilience and pharmacy performance.

## 5. Discussion

This study developed a conceptual model to evaluate the interrelationships among resilience, pharmacy performance, and patient satisfaction in urban outpatient pharmacies. The findings revealed that pharmacy resilience is directly correlated with both operational performance and patient satisfaction. The results of this study help pharmacies to utilize their resources more efficiently in order to adapt to a changing environment and evolving consumer needs, ultimately leading to enhanced levels of patient satisfaction.

The findings demonstrated a statistically significant and strong association between resilience and pharmacy performance (R = 0.642), confirming that resilience is not merely a theoretical construct but a functional capacity that translates directly into enhanced operational outcomes. Moreover, the significant correlation between resilience and patient satisfaction (R = 0.496), and between performance and satisfaction (R = 0.334), underscores the importance of organizational resilience not only in ensuring continuity of care but also in maintaining trust and satisfaction among service recipients. These findings reinforce the emerging view that resilience is integral to both system stability and patient-centered care delivery. This aligns with prior research highlighting that organizational resilience becomes evident in unusual conditions such as shocks, crises (e.g., COVID-19), and other disruptions (23). Consequently, resilience is closely linked to organizational survival, as it enables entities to withstand crises, absorb shocks, and navigate various challenges effectively (24). Organizational resilience and financial performance can serve as proxies for measuring overall organizational performance (25). Moreover, organizational resilience impacts various dimensions of business performance, including economic-financial outcomes, customer satisfaction, and internal processes as well as learning. The distinct effects of resilience on business performance provide managers with valuable guidance for resource allocation, thereby supporting the employer's interests (26). Findings from previous research on resilience have shown its influence on multiple performance metrics, including organizational survival, operational effectiveness, profitability measures, and sales growth (27, 28). It has been established that resilience affects business performance, indicating that resilient companies consistently outperform their non-resilient counterparts (29). Organizational resilience promotes effective responses to environmental change and supports the development of diverse organizational capabilities (30). Organizational resilience involves introducing new capabilities and increasing the ability to monitor and generate new opportunities for innovation. Researchers argue that organizational resilience is a dynamic process through which members adapt positively, enhancing their organization's competitiveness in the aftermath of a crisis. Resilience plays a crucial role in enhancing organizational effectiveness during challenging circumstances by enabling individuals, teams, and organizations to navigate and adapt effectively to unforeseen events (31, 32).

The success or failure of a company depends on its performance. Organizational performance can be challenging due to different standards including economic viability, environmental responsiveness, and long-term sustainability (33). Human resources play a crucial role in influencing the performance of companies and are recognized as a key element in ensuring organizational resilience (34). While the COVID-19 pandemic has posed an unprecedented crisis in both magnitude and industry-wide impact, it is not the first disruptive event to influence the performance of companies (35). Prolonged economic downturns negatively affect companies, leading to declining sales, reduced profits, financial strain, and challenges in meeting supplier obligations. Nonetheless, certain companies manage to achieve considerable success despite experiencing a crisis (36). Therefore, to strengthen their ability to maintain business continuity, particularly in times of crisis such as a pandemic, businesses must enhance their resilience capabilities. This involves reinforcing both proactive resilience, which anticipates and prepares for potential disruptions, and reactive resilience, which enables organizations to respond and recover from such challenges (37). In emerging economies, well-managed companies perform better during and in the aftermath of global crises. Firms that demonstrate high-quality management and pursue innovation are more likely to enhance their performance in times of crisis (38). In line with the results of this investigation, previous studies indicate that the resilience of an organization exerts a positive influence on a company's performance level (32). Organizational resilience stems from strong performance, making it essential for management to effectively utilize resources and capitalize on opportunities in sales and service (37).

The relationship between resilience and pharmacy performance has garnered increasing attention, particularly considering recent global health crises. Resilience within pharmacy practice, both at the individual and institutional levels, is recognized as a determinant of sustained service delivery under stress, directly influencing performance outcomes such as medication availability, patient counseling quality, and operational continuity. Studies suggest that pharmacies with higher adaptive capacity, proactive resource planning, and organizational support systems demonstrate greater responsiveness to surges in demand and disruptions in supply chains, resulting in improved patient satisfaction and service reliability (39, 40). This evidence reinforces the notion that embedding resilience-building strategies within pharmacy operations is not only beneficial but also essential for ensuring robust performance during crises.

Importantly, patient satisfaction was also positively associated with both resilience and performance, suggesting that the benefits of resilience extend beyond internal organizational metrics and encompass patient-perceived quality of care. This further supports the emerging paradigm that views resilience as a multidimensional construct with systemic as well as interpersonal implications (7, 41). Partial correlation analysis, which controlled for potential confounders, confirmed the direct and robust nature of the observed relationships. The relationship between resilience and performance remained strong (R = 0.542), as did the correlation between resilience and patient satisfaction (R = 0.496). This is consistent with prior research that underscores the positive and significant influence of resilience on customer loyalty with respect to both behavioral and attitudinal dimensions, as evidenced by studies reporting a positive relationship between resilience and sustainability practices in relation to customer loyalty (13).

The extent of organizational resilience and survival can be influenced by trust and strategic flexibility (37). Prior research has emphasized the critical role of operational resilience in fostering customer loyalty (42). Moreover, the effects of responsiveness and service quality on customer loyalty have been examined as distinct constructs, with studies emphasizing that customers are fundamental assets for organizational continuity, particularly within the MSMEs sector (13).

The results indicate that managerial age significantly affects key outcomes, highlighting its influence on both operational effectiveness and patient-centered care. Pharmacy managers aged above 60 exert a significant impact on these outcomes, thereby underscoring the role of managerial age in shaping both operational effectiveness and patient-centered care. This is consistent with previous studies that have demonstrated a significant relationship between respondents' age and various dimensions, including decision-making, human resource management, and overall managerial effectiveness (43). Additionally, pharmacies managed by male individuals reported higher patient satisfaction score, suggesting a potential gender-related variation in perceived service quality or communication style. Male healthcare providers were consistently rated highly for professionalism and privacy, reflecting societal expectations that position men within roles of technical expertise and authority (44). This perception is reinforced by social role theory, which emphasizes the traditional linkage of male gender roles with assertiveness and competence in leadership contexts. This is further evidenced by the significant positive impact of attributes such as professionalism and privacy on patient satisfaction, especially in domains requiring accuracy and discretion and most notably among male providers (45).

This study underscores the potential influence of geographic and locational determinants, including demographic characteristics, access to resources, and regional healthcare infrastructure on both operational and patient-centered outcomes. Findings revealed that resilience, pharmacy performance, and patient satisfaction were comparatively higher in pharmacies situated in the northern and western regions of Tehran. This indicates that geographic context contributes to shaping both operational dynamics and patient perceptions. The surrounding physical environment of healthcare facilities, such as cleanliness, noise levels, and overall ambiance, can significantly influence patient comfort and perception of care quality, thereby affecting overall satisfaction (46, 47). Patients from areas with greater socioeconomic disadvantage often report lower levels of satisfaction, attributable to restricted access to resources, transportation barriers, and systemic biases encountered within the healthcare system (48).

Pharmacy resilience plays a vital role in safeguarding and enhancing public health. Resilient pharmacies can identify and manage risks, maintain the continuous delivery of high-quality services, while learning from past experiences. Through these mechanisms, they effectively meet patients' pharmaceutical needs and contribute to enhanced patient satisfaction. Ultimately, organizational resilience enables adaptive responses to fluctuating demands in dynamic environments and strengthens organizations’ capacity to manage unexpected disruptions (13). By improving operational efficiency and overall performance, resilience supports the meeting of customer expectations and the cultivation of loyalty. Furthermore, its positive impact on performance ultimately leads to greater customer satisfaction and long-term loyalty.

### 5.1. Conclusions

This research aims to investigate the correlation between resilience, defined in terms of readiness, response, recovery, learning and growth, and pharmacy performance measured by financial performance, pharmacy process, and growth, as well as patient satisfaction accessed through pharmacy facilities, and the dimension of service quality. Based on a comprehensive review of literature and prior studies, the research model was formulated to conceptualize the underlying theoretical constructs and identify the existing research gap. The findings confirmed a positive and significant relationship between resilience, pharmacy performance, and patient satisfaction in pharmacy. Overall, this study offers a practical and evidence-based framework to guide resilience-building strategies in pharmacy settings, particularly in preparation for future public health emergencies. By integrating resilience with performance and patient satisfaction, the proposed model shows promise for informing managerial decision-making, shaping training protocols, and supporting policy development within pharmacy systems.

### 5.2. Limitations

This study has several noteworthy limitations. First, the model was developed within a specific national context, which may limit its generalizability to other settings. Second, reliance on self-reported data introduces the possibility of response bias. Furthermore, data collection was constrained by limited access to public and hospital pharmacies, as well as reluctance of some respondents to disclose income-related information. Another important limitation of the study was the potential for recall bias. Given the considerable time that has elapsed since the COVID19 pandemic, the interview questions were deliberately designed with a broad scope to ensure comprehensive coverage of the relevant issues. These factors may have reduced the representativeness of the sample and introduced potential bias into performance-related findings. Future research should aim to validate the model across diverse geographic and policy contexts and incorporate longitudinal data to capture the evolution of resilience over time.

## Data Availability

The dataset presented in the study is available on request from the corresponding author during submission or after its publication.
